# Plasmacytoid Dendritic Cells Are Crucial in *Bifidobacterium adolescentis*-Mediated Inhibition of *Yersinia enterocolitica* Infection

**DOI:** 10.1371/journal.pone.0071338

**Published:** 2013-08-20

**Authors:** Alexandra Wittmann, Ingo B. Autenrieth, Julia-Stefanie Frick

**Affiliations:** 1 Institute for Medical Microbiology and Hygiene, University Hospital Tübingen, Tübingen, Germany; 2 German Centre for Infection Research, University of Tübingen, Tübingen, Germany; Charité, Campus Benjamin Franklin, Germany

## Abstract

In industrialized countries bacterial intestinal infections are commonly caused by enteropathogenic *Enterobacteriaceae*. The interaction of the microbiota with the host immune system determines the adequacy of an appropriate response against pathogens. In this study we addressed whether the probiotic *Bifidobacterium adolescentis* is protective during intestinal *Yersinia enterocolitica* infection. Female C57BL/6 mice were fed with *B. adolescentis*, infected with *Yersinia enterocolitica*, or *B. adolescentis* fed and subsequently infected with *Yersinia enterocolitica. B. adolescentis* fed and *Yersinia* infected mice were protected from *Yersinia* infection as indicated by a significantly reduced weight loss and splenic *Yersinia* load when compared to *Yersinia* infected mice. Moreover, protection from infection was associated with increased intestinal plasmacytoid dendritic cell and regulatory T-cell frequencies. Plasmacytoid dendritic cell function was investigated using depletion experiments by injecting *B. adolescentis* fed, *Yersinia* infected C57BL/6 mice with anti-mouse PDCA-1 antibody, to deplete plasmacytoid dendritic cells, or respective isotype control. The *B. adolescentis*-mediated protection from *Yersinia* dissemination to the spleen was abrogated after plasmacytoid dendritic cell depletion indicating a crucial function for pDC in control of intestinal *Yersinia* infection. We suggest that feeding of *B. adolescentis* modulates the intestinal immune system in terms of increased plasmacytoid dendritic cell and regulatory T-cell frequencies, which might account for the *B. adolescentis*-mediated protection from *Yersinia enterocolitica* infection.

## Introduction

Infection with *Yersinia enterocolitica* e.g. by ingestion of contaminated food or drinking water can cause severe diarrhea, enterocolitis, and mesenteric lymphadenitis [1], [2]. *Yersinia enterocolitica* is a facultative anaerobic, pleomorphic, gram-negative rod that belongs to the family of *Enterobacteriaceae* and its enteropathogenicity is associated with the presence of a 70-kb virulence plasmid (pYV) that encodes a type three secretion system, translocated effector proteins, and the trimeric autotransporter *Yersinia* adhesin A (YadA) [3], [4].

Several studies demonstrate that the host's intestinal microbiota is crucial in defining the host's susceptibility towards intestinal infections. This is demonstrated by the significant influence of antibiotic treatment on the composition of the intestinal microbiota, in both, human subjects [5], [6], [7], [8] and mice [9], [10] where increased susceptibility towards enteropathogenic bacteria was shown [11], [12]. The intestinal microbiota is thought to shape the innate immune system in different ways. It was demonstrated that antibiotic treatment of mice and subsequent alterations of the intestinal microbiota notably down-regulate the expression of Reg3γ, a secreted C-type lectin which kills gram-positive bacteria including e.g. antibiotic-resistant bacteria such as vancomycin resistant *Enterococcus* (VRE) [13]. The secretion of Reg3γ could be restored via stimulation of intestinal TLR4 thereby boosting the innate immune resistance of antibiotic-treated mice against infections with VRE [13]. In addition, antibiotic-induced disruption of the intestinal microbiota enhances the susceptibility of human hosts to infections with nontyphoidal *Salmonellae* [14], and is a prerequisite for infection of mice with *Salmonella typhimurium* [15], [16].

Probiotics are defined as live microorganisms that, when administered in adequate amounts, confer a beneficial effect on the health of the host [17]. E. g. *Lactobacillus rhamnosus* GG, *Bifidobacterium bifidum, Streptococcus thermophilus* and *Saccharomyces boulardii* revealed a beneficial effect on children with rotavirus infections [18], [19], [20]. Furthermore, several studies summarized by Nomoto *et al.* report a decrease in the incidence of antibiotic-induced diarrhea by administration of *Saccharomyces boulardii*, *Lactobacillus rhamnosus* GG, *Bifidobacterium longum* and *Enterococcus faecium* [21].

Dendritic cells (DCs) are essential initiators of immunity and link innate to adaptive antimicrobial immune responses [22]. In order to fulfill these tasks, first DC need to sample intestinal antigens. These are acquired by DC either in Peyer's patches, where M cells deliver luminal content by transcytosis [23], [24], or DC can sample actively by extending dendrites into the lumen without disrupting epithelial tight junctions due to the expression of CX_3_CR1 [25]. However, this subset of DC was demonstrated to be non-migratory whereas CD103-expressing intestinal DC migrate to the mesenteric lymph nodes in a big way after antigen acquisition, for which they rely on other cell types [26]. In the intestine conventional DC (cDC) are made up by these two subsets which both can additionally express CD11b [26] and occur in the lamina propria as well as the Peyer's patches [25], [27]. In addition to cDC, plasmacytoid DC exist and are defined by their intermediate expression of CD11c and additional expression of PDCA-1 and B220 [28]. They are found to a minor extent in secondary lymphoid organs [29] and the lamina propria but occur mainly in the intraepithelial compartment [28]. In response to TLR9 and TLR7 signalling pDCs produce high amounts of type I IFNs [26]. In addition, pDCs are thought to be of key importance for the regulation of tolerance and are considered to be inducers of regulatory T cells [30], [31], [32].

In previous work we identified a probiotic *B. adolescentis* strain that attenuated the course of *Y. enterocolitica* infection in mice by reducing clinical symptoms, dissemination of *Yersinia*, and *Y. enterocolitica*-induced mucosal inflammation [33]. In this study we demonstrate that feeding of viable *B. adolescentis* was associated with an increased number of PDCA-1-positive pDCs in the intestine and an attenuated course of *Y. enterocolitica* infection as indicated by reduced clinical symptoms and reduced dissemination of *Yersinia.*

## Materials and Methods

### Mice

6–10****weeks old female C57BL/6 mice were purchased from Harlan Laboratories, after transfer mice were housed under specific pathogen free conditions in isolated ventilated cages. Animal experiments were reviewed and approved by an appropriate institutional review committee (Genehmigung H2/05 Regierungspräsidium Tübingen).

### Cultivation of *B. adolescentis* and Oral *Yersinia* Infection of Mice

For feeding experiments, *Bifidobacterium adolescentis* Reuter 1963 (ATCC 15705) was anaerobically incubated in soy broth containing beef liver at 37°C for 48****h, then transferred into *Bifidobacteria* medium (formula according to M58-medium Leibniz Institute *DSMZ-German Collection of Microorgansims and Cell Cultures*) and cultivated under same conditions for additional 48****h. Bacteria were twice washed in phosphate buffered saline (PBS) (PAA) and thereafter resuspended in aseptic drinking water. 48****h prior to *B. adolescentis* feeding mice obtained drinking water containing streptomycin (20****g/l Sigma). Mice were intragastrically infected with 5×10^8^ plasmid harboring *Y. enterocolitica* WA-314 serotype O8 [34] as previously described [33]. Body weight development of mice was monitored daily, four days post infection mice were sacrificed by carbon dioxide asphyxiation, and subsequently the complete intestine, Peyer's patches, and the spleen were removed.

### Antibiotic Treatment

Prior to *B*. *adolescentis* feeding, streptomycin containing drinking water (20****g/l Sigma) was administered to mice for 48****h in order to facilitate efficient *B. adolescentis* colonization. For *B. adolescentis* depletion experiments, mice received vancomycin (1****g/l Hexal) and metronidazole (1****g/l Sigma) containing drinking water for 48****h.

### Determination of Colony Forming Units (CFU) of *Yersinia*

Fecal samples, Peyer's patches, and spleens were scaled. Tissue samples were homogenized by extruding through 40 µm cell strainer (BD Falcon). After, complete samples were resuspended in aseptic PBS, serial diluted until a factor of 10^−4^, plated on CIN plates (Oxoid), and incubated for 48****h at 27°C. CFU were calculated per gram samples. The detection limit of CFU of Yersinia was 10.

### Isolation of Lamina Propria and Intraepithelial Leukocytes

After removal of the complete intestine, Peyer's patches were abscised, the gut was opened longitudinally, and flushed thoroughly with phosphate buffered saline (Gibco) containing fetal calf serum (1 w/v; Sigma) (PBS/FCS). Intestinal epithelial cells were separated from the lamina propria by incubation in 1****mM dithiothreitol (DTT; AppliChem) and 1****mM ethylenediaminetetraacetic acid (EDTA) containing PBS under slow rotations. The soluble epithelial fraction was washed in PBS/FCS. Remaining intestinal tissue was washed in PBS/FCS, cut thoroughly, subsequently transferred into digestion solution (VLE RPMI 1640, Biochrom; 200 U/ml collagenase, Sigma; 5 U/ml DNase, Roche; 50 µM β-mercaptoethanol, AppliChem; 5% FCS, Sigma; 2****mM glutamine, 2500 U/ml penicillin, 2000 µg/ml streptomycin, GIBCO) and incubated for 60****min under slow rotations. Lamina propria – and epithelial cell suspensions were centrifuged, resuspended in 40% Easycoll (Biochrom) dilution, cautiously layered on 70% Easycoll dilution, and then centrifuged. Leukocytes were extracted from the interlayer and washed.

### Flow Cytometry Analysis of Lamina Propria and Intraepithelial Leukocytes

Isolated lamina propria (lp) and intraepithelial (ie) leukocytes were incubated in FC Receptor Block (Fcγ III/II receptor; clone 2.4G2). Surface markers were stained with antibody concentrations of 1 µl per 1×10^6^ cells for 25****min at 4°C and subsequently washed in PBS/FCS. Applied antibodies were Fluorescein-isothiocyanate (FITC) labeled anti-mouse CD3ε clone 145-2C11, anti-mouse CD19 clone 1D3, anti-mouse CD49b/Pan-NK clone DX5, peridinin-chlorophyll-protein complex (PerCP) labeled anti-mouse CD4 clone RM4-5, allophycocyanin (APC) labeled anti-mouse CD11b clone M1/70, CD45R/B220 clone RA3-6B2, and biotinylated anti-mouse CD11c clone HL3 (BD Bioscience), and phycoerythrin (PE) labeled anti-mouse PDCA-1 clone JF05-1C2.4.1 (Miltenyi Biotec). Specimens containing biotinylated antibodies were thereafter incubated with 1 µl of second step fluorescence conjugate (Streptavidin-PercP; BD Bioscience) for 15****min at 4°C. Next cells were washed in PBS/FCS and then resuspended in PBS/FCS containing 1.5% paraformaldehyde. For intracellular staining cells were incubated in Cytofix/Cytoperm™ (BD Bioscience) for 20****min at 4cC then washed in permeabilizing wash buffer (PWB)(PBS, 0.1% saponin (Sigma) and 3% FCS). Thereafter permeabilized cells were stained with 0.5 µl PE labeled anti-mouse FoxP3 clone FJK-16s antibody per 1×10^6^ cells for 20****min at 4°C in PWB. Afterwards cells were first washed in PWB, then in PBS/FCS, and finally resuspended in PBS/FCS containing 1.5% paraformaldehyde. Specimens were measured on a FACSCalibur^TM^ or LSRFortessa (BD Bioscience) using BD CellQuest Pro™ or BD FACSDIVA™ software, respectively. Data were analyzed with WinMDI Software Version 2.8 (Joe Trotter) or FlowJo 7.6.4 (TreeStar Inc.). Forward and Side Scatter were used to exclude dead cells, cell debris and cell aggregates of analysis. DCs were analyzed by electronically gating on Lin^−^ (CD3ε, CD19, DX5) negative and CD11c-positive (CD11c^+^) cells, as indicated in respective figures. For analyses of FoxP3 expression lamina propria leukocytes were gated on CD3ε and CD4-expressing T cells, as indicated.

### Plasmacytoid Dendritic Cell Depletion

Mice were *B. adolescentis* fed and *Yersinia* infected as earlier described. In addition, mice were 1.5****days prior to and 0.5****days post *Yersinia* application intravenously injected with 150 µg of functional grade pure anti-mouse PDCA-1 antibody clone JF05-1C2.4.1 (Miltenyi Biotec) or functional grade purified Rat IgG2b isotype control (eBioscience).

### Statistics

Statistical significances were calculated using unpaired t-test, when variances were statistically significantly different unpaired t-test with Welch's correction was used instead. For experiments with more than two investigated groups statistical significances were calculated using One-way analysis of variance followed by Tukey's Multiple Comparison Test. P values smaller than 0.05 were considered significant.

## Results

### The *Bifidobacterium adolescentis*-Mediated Protection from *Yersinia enterocolitica* Infection is Associated with an Increased Proportion of PDCA-1-Positive pDCs

First we analyzed the effect of *B. adolescentis* feeding on the intestinal mucosal immune system, in particular on the composition of intraepithelial (ie) DC and the lamina propria (lp) DC subpopulations and the course of *Yersinia enterocolitica* infection. Therefore female C57BL/6 mice were fed with (i) PBS as mock control (M), (ii) viable *B. adolescentis* (B), (iii) infected with 5×10^8^ CFU *Yersinia enterocolitica* (Y) or (iv) fed with *B. adolescentis* and subsequently infected with *Yersinia enterocolitica* (BY).

**Figure 1:**
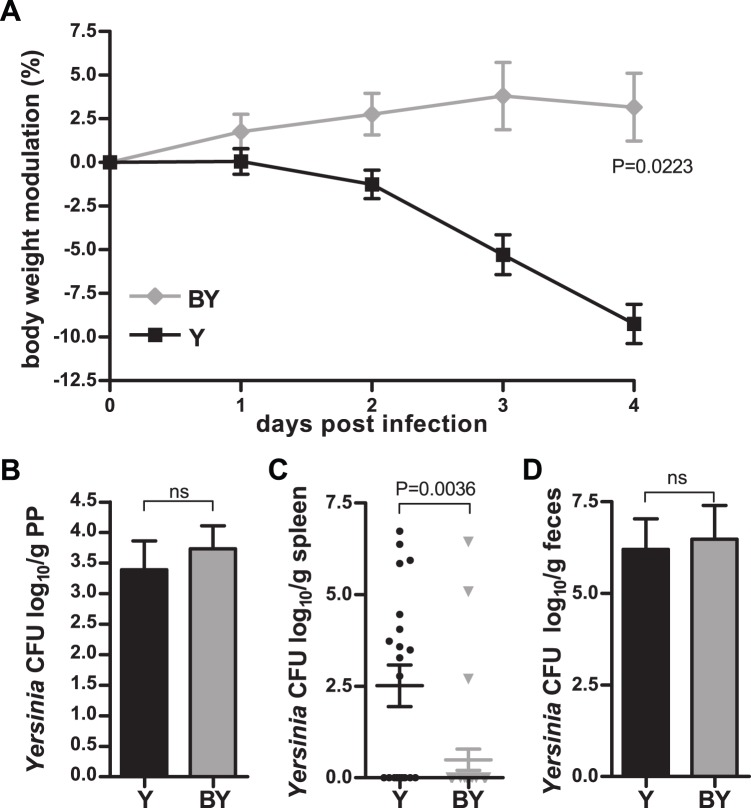
*B. adolescentis* Feeding Prevents From Weight Loss and Dissemination of *Yersinia* to the Spleen. (**A**) Graph represents percent modulation of initial body weight over four days of *Yersinia* infected mice (Y, black line and squares) and *B. adolescentis* fed and *Yersinia* infected mice (BY, gray line and diamonds). Colony forming units (CFU) of *Yersinia* in log_10_ per gram Peyer's patches (PP) (**B**), spleen (**C**), or feces (**D**) of *Yersinia* infected mice (Y) and *B. adolescentis* fed and *Yersinia* infected mice (BY). Data represent mean and SEM of at least 8 mice.

BY mice (3.2%±7%) were significantly protected in terms of weight loss as compared to Y mice (−9.3%±3.4%; p = 0.0223) (**Fig. 1A**). Determination of CFU of *Yersinia* in the Peyer's patches revealed no differences between BY (log_10_ 3.7±1.8) and Y (log_10_ 3.4±1.8) mice (**Fig. 1B**). However, the BY mice were protected from dissemination of *Yersinia* during infection as indicated by the significantly reduced CFU of *Yersinia* in the spleens of *B. adolescentis* fed *Y. enterocolitica* infected mice (log_10_ 0.5±1.6) when compared to Y mice (log_10_ 2.5±2.5; p = 0.0036) (**Fig. 1C**). We were able to exclude that feeding of *B. adolescentis* reduced the number of intestinal enteropathogenic *Yersinia* in the BY mice, since BY (log_10_ 6.5±0.9) and Y mice (log_10_ 6.2±0.8) harbored comparable counts of *Yersinia* in the intestine (**Fig. 1D**).

These results are in line with previous results of our group demonstrating protective effects of *B. adolescentis* in both, inflammatory as well as infectious intestinal diseases [33].

**Figure 2:**
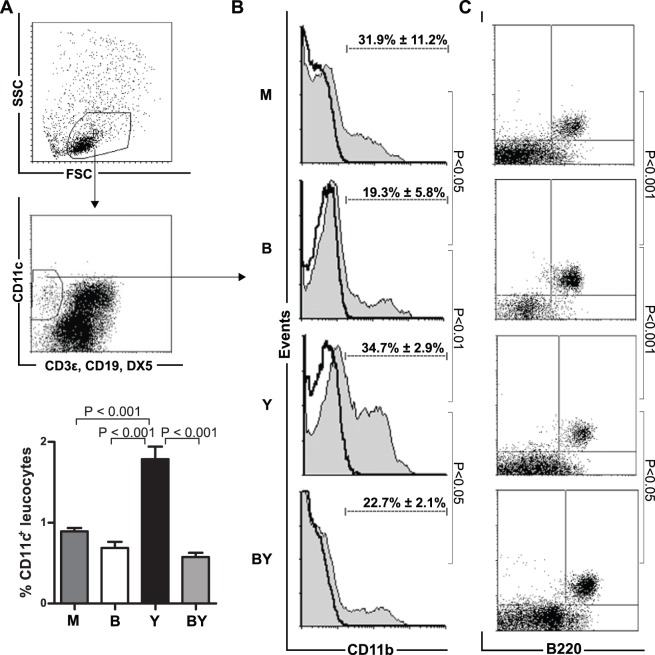
B. adolescentis Feeding Results in Increased Plasmacytoid DC Frequency. (**A**) Intraepithelial dendritic cells (ieDC) were analyzed by gating on linage negative (CD3ε, CD19, DX5) and CD11c-positive cells, as demonstrated in exemplarily depicted dot plots. Graph represents mean percentage ± SEM of CD11c^+^ intraepithelial leukocytes of untreated mock (M, dark gray bar), *B. adolescentis* fed (B, white bar), *Yersinia* infected (Y, black bar), as well as *B. adolescentis* fed and *Yersinia* infected mice (BY, gray bar). (**B**) Histograms represent mean percentage ± SD of CD11b-expressing conventional ieDCs (solid black line) and respective fluorescence minus one control (gray filled histogram). (**C**) Dot plots indicate mean percentage ± SD of B220 and PDCA-1-expressing plasmacytoid ieDCs of mock (M), *B. adolescentis* fed (B), *Yersinia* infected (Y), as well as *B. adolescentis* fed and *Yersinia* infected mice (BY). Data represent five mice per group.

Analysis of ieDC frequency in the intestine of differently treated mice, exhibited a significant increase in the percentage of ieDCs in Y mice (1.8%±0.3%) as compared to remaining groups (M (0.9%±0.1%) p<0.001, B (0.7%±0.2%) p<0.001, BY (0.6%±0.1%) p<0.001) (**Fig. 2A** **,** **Table 1**). This finding was confirmed by total numbers of dendritic cells which were present in different groups (M (1.9×10^4^±1.3×10^4^) p<0.001, B (3.0×10^4^±0.8×10^4^) p<0.01, Y (5.7×10^4^±0.9×10^4^), BY (1.9×10^4^±1.3×10^4^) p<0.001) (**Table 1**).

**Table 1:** Cell Counts of Total Intraepithelial DCs, Conventional, and Plasmacytoid DCs.

Colonization	CD11c^+^ cells	Conventional DC	Plasmacytoid DC
	cell count ×10^4^	cell count ×10^3^	cell count ×10^3^
**M**	1.9±1.3	6.1±5.6	1.7±0.5
**B**	3.0±0.8	5.9±1.2	7.7±2.4^c^
**Y**	5.7±0.9^a^	19,9±10^b^	4.8±2.7
**BY**	1.9±1.3	4.4±3.5	3.5±3

Numbers indicate mean cell counts ± SD of untreated mock (M), *B. adolescentis* fed (B), *Yersinia* infected (Y), as well as *B. adolescentis* fed and *Yersinia* infected mice (BY) mice. ^a^ M vs. Y p<0.001, B vs. Y p<0.01, BY vs. Y p<0.001; ^b^ M vs. Y p<0.05, B vs. Y p<0.05, BY vs. Y p<0.01; ^c^ Mock vs. B p<0.01, and B vs. BY p<0.05. Data represent five mice per group.

Next the ieDC subset composition of mice with or without *B. adolescentis* feeding and with or without *Yersinia* infection, respectively, was investigated. Since great parts of intestinal conventional (c) DC, positive for either CX_3_CR1 or CD103, express CD11b [26] we used for the sake of convenience this marker to determine cDC. Results demonstrated that feeding of *B. adolescentis* led to a significantly reduced percentage of CD11c^+^CD11b^+^ ieDCs in both B and BY mice (M (31.9%±11.2%) vs. B (19.3%±5.8%) p<0.05, Y (34.7%±2.9%) vs. BY (22.7±2.1%) p<0.05, B vs. Y p<0.01).

No significant differences in the percentage frequency of CD11c^+^CD11b^+^ ieDC of Y mice were observed as compared to M mice (**Fig. 2B**). However, analysis of total numbers indicated a significant increase of CD11c^+^CD11b^+^ ieDCs only in Y mice (Y (19.9×10^3^±10×10^3^) as compared to all other groups (M (6.1×10^3^±5.6×10^3^) p<0.05, B (5.9×10^3^±1.2×10^3^) p<0.05, BY (4.4×10^3^±3.5×10^3^) p<0.01) (**Table 1**). The differences between absolute numbers of CD11c^+^CD11b^+^ ieDCs between M mice and Y mice might be caused by an increased influx of CD11c^+^ leukocytes to the *Yersinia* infected intestine (**Table 1**).

Analysis concentrating on the percentage of CD11c^+^B220^+^PDCA-1^+^-expressing plasmacytoid (p)DCs demonstrated, however, that feeding of *B. adolescentis* resulted in a significantly increased percentage of pDCs in both B mice and BY mice (M (9.2%±1.2%) vs. B (25.7%±4.8%) p<0.001, Y (8.3%±2.5%) vs. BY (18.2%±8.4%) p<0.05, B vs. Y p<0.001) (**Fig. 2B**). The total number of pDCs was significantly increased in B mice (7.7×10^3^±2.4×10^3^) but not in the other groups (M (1.7×10^3^±0.5×10^3^) p<0.01, Y (4.8×10^3^±2.7×10^3^), BY (3.5×10^3^±3×10^3^) p<0.05).

The percentage frequency of pDC in BY mice might lead to the conclusion that BY mice need to have higher absolute cell counts as Y mice do. However, due to the fact that Y mice have in general a high absolute number of dendritic cells these mice even have with a low percentage frequency of pDC a high absolute frequency. Nevertheless, in Y mice the ratio between pDC and cDC is shifted in favor of cDC whereas in BY mice it is more balanced (Fig. 2, **Table 1**).

**Figure 3:**
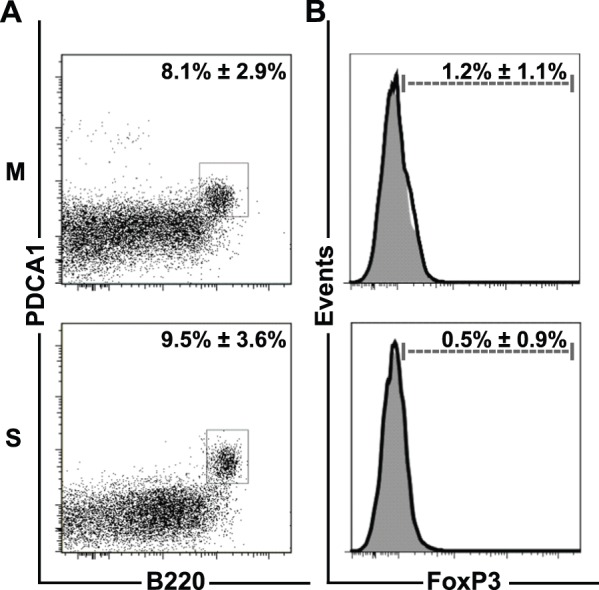
Streptomycin Treatment does Neither Induce Intraepithelial pDCs nor Lamina Propria T_reg_ Cells. Mean percentage ± SD of (**A**) intraepithelial B220^+^PDCA-1^+^ plasmacytoid DCs and (**B**) lamina propria FoxP3-expressing regulatory T cells (solid black line) and fluorescence minus one control (gray filled histograms) of untreated mock (M) and streptomycin (S) treated mice. Intraepithelial plasmacytoid cells and lamina propria CD4^+^ T cells were gated as indicated in Fig. 2 and Fig. 5, respectively. Data represent at least four mice per group.

**Figure 4:**
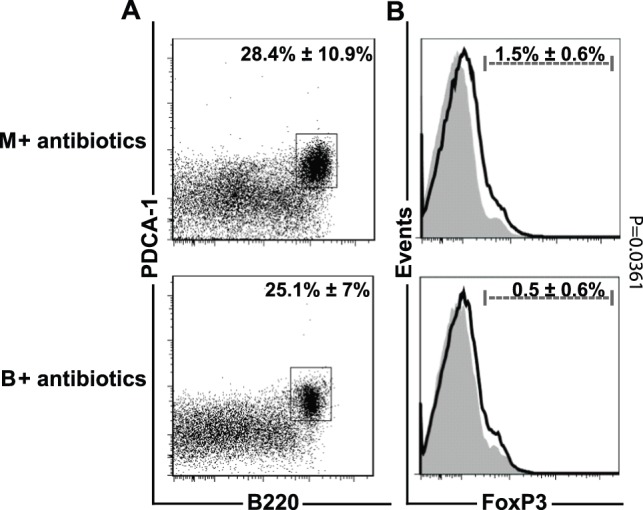
*B. adolescentis* Depletion Results in Comparable Frequencies of Intraepithelial pDCs and Lamina Propria T_reg_ Cells. Mean percentage ± SD of (**A**) intraepithelial B220^+^PDCA-1^+^ plasmacytoid DCs and (**B**) lamina propria FoxP3-expressing regulatory T cells (solid black line) and fluorescence minus one control (gray filled histograms) of vancomycin and metronidazole treated mock (M+ antibiotics) and *B. adolescentis* (B+ antibiotics) (B) fed mice. Intraepithelial plasmacytoid cells and lamina propria CD4+T cells were gated as indicated in Fig. 2 and Fig. 5, respectively. Data represent five mice per group.

The analysis of the lpDCs gave comparable results: a significant decrease of CD11c^+^CD11b^+^ lpDCs (M (70%±5.1%) vs. B (58.6%±8.2%) p<0.05, Y (70.7%±3.8%) vs. BY (57.7%±9.3%) p<0.05, M vs. BY p<0.05, B vs. Y p<0.05) and a significant increase in CD11c^int^B220^+^ and CD11c^int^PDCA-1^+^ plasmacytoid (p) DCs in *B. adolescentis* fed mice regardless of additional infection with *Yersinia* or not (CD11c^int^B220^+^: M (6.8%±1.3%) vs. B (16.8%±1.4%) p<0.001, Y (6.7%±2%) vs. BY (17.2%±3.2%) p<0.001, M vs. BY p<0.001, B vs. Y p<0.001; CD11c^int^PDCA-1^+^: M (2.9%±0.7%) vs. B (9.3%±1.2%) p<0.001, Y (2.9%±1.5%) vs. BY (6.7%±1.4%) p<0.001, M vs. BY p<0.001, B vs. Y p<0.001, B vs. BY p<0.001). (**Fig. S1, Table S1, Table S2**). To exclude that the enhanced frequency of pDCs in B and BY mice was due to streptomycin treatment prior to *B. adolescentis* administration C57BL/6 mice were treated with streptomycin (S) only and the frequency of intraepithelial pDCs was analyzed. Application of streptomycin did not result in an increase frequency of pDCs (M (8.1%±2.9%, 1.1×10^4^±0.6×10^4^); S (9.5%±3.6%, 1.2×10^4^±0.5×10^4^), therefore we can exclude the influence of streptomycin treatment or streptomycin caused alterations of the intestinal microbiota on the recruitment or onsite development of pDCs (**Fig. 3A** **,** **Table 2**). In order to demonstrate that the increased frequency of intraepithelial pDCs in B and BY mice is a result of *B. adolescentis* feeding, mice were either fed with *B. adolescentis* for 6****days or not and subsequently treated with vancomycin and metronidazole for 2****days. After 5****weeks the frequency of intraepithelial pDCs was determined. The antibiotics-induced reduction of *B. adolescentis* in the intestine resulted in comparable numbers of pDCs in antibiotic treated mock (28.4%±10.9%, 16.8×10^3^±1.3×10^3^) as well as *B. adolescentis* fed and antibiotic treated mice (25.1%±7%, 9.3×10^3^±3.1×10^3^). However, it remains elusive whether this is a direct effect of *B. adolescentis* or a secondary effect on the intestinal microbiota (**Fig. 4A** **,** **Table 3**).

**Table 2:** Cell Counts of Intraepithelial Plasmacytoid DCs, and Lamina Propria FoxP3^+^ T_reg_ Cells.

Treatment	Plasmacytoid DC	CD4^+^ FoxP3^+^cells
	cell count ×10^4^	cell count ×10^1^
**M**	1.1±0.6	8.7±6.5
**S**	1.2±0.5	0.7±0.1

Numbers indicate mean cell counts±SD of untreated mock (M) and streptomycin (S) treated mice. Data represent at least four mice per group.

**Table 3:** Cell Counts of Intraepithelial Plasmacytoid DCs, and Lamina Propria FoxP3^+^ T_reg_ Cells.

Treatment	Plasmacytoid DC	CD4^+^ FoxP3^+^cells
	cell count ×10^3^	cell count ×10^3^
**M + antibiotics**	16.8±1.3	3.4±2.5^a^
**B + antibiotics**	9.3±3.1	0.3±0.3

Numbers indicate mean cell counts±SD of vancomycin and metronidazole treated mock (M)and *B. adolescentis* (B) fed mice. Data represent five mice per group. ^a^ p = 0.00494.

### *B. adolescentis*-Mediated Protection is Associated with an Increase in FoxP3^+^ Regulatory T Cells

Plasmacytoid DCs are described to induce both protective adaptive immunity as well as immune tolerance, depending on their localization and state of activation [22]. Moreover, this subset is associated with the induction of FoxP3^+^CD4^+^CD25^+^-expressing regulatory T cells (T_reg_ cells) [35] therefore we next analyzed the frequency of CD3ε^+^CD4^+^ cells and of FoxP3^+^CD4^+^ T_reg_ cells.

**Figure 5:**
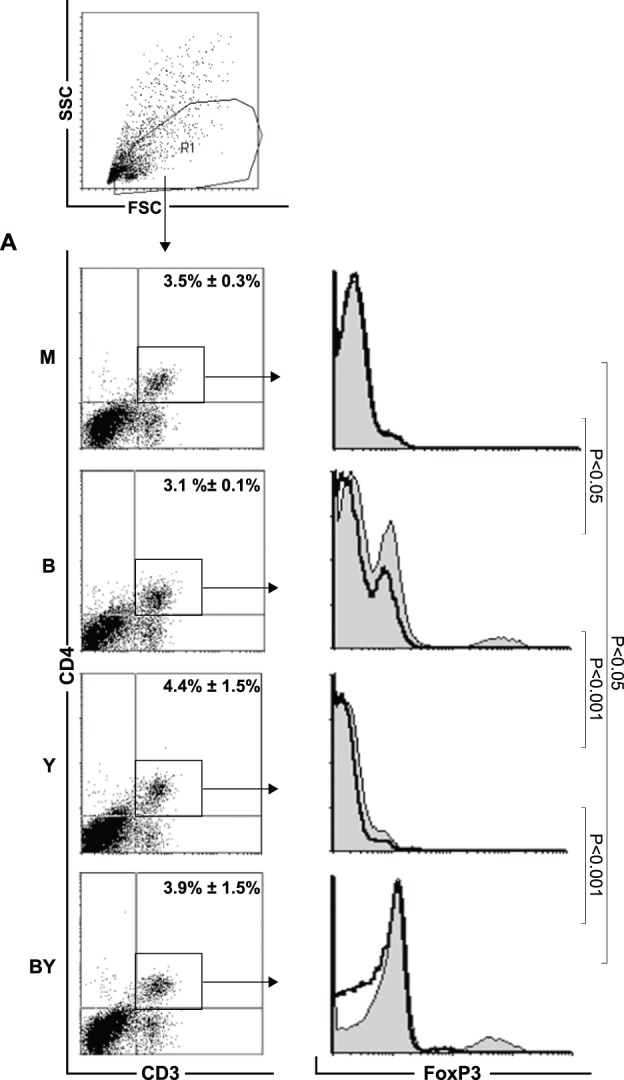
*B. adolescentis* feeding increases Frequencies of Lamina Propria T_reg_ Cells Independent from State of Inflammation. Mean percentage ± SD of lamina propria (A) CD3ε^+^CD4^+^ T cells and (B) FoxP3-expressing regulatory CD3ε^+^CD4^+^ T cells (solid black line) and fluorescence minus one control (gray filled histograms) of mock (M), *B. adolescentis* fed (B), *Yersinia* infected (Y), as well as *B. adolescentis* fed and *Yersinia* infected mice (BY) mice. Cells were gated as indicated. Data represent at least five mice per group.

Independent of treatment, mice exhibited no differences in the percentage and absolute number of CD4^+^ T cells (M (3.5%±0.3%, 1.3×10^5^±0.7×10^5^), B (3.1%±0.1%, 1×10^5^±0.4×10^5^), Y (4.4%±1.5%, 0.9×10^5^±0.5×10^5^), BY (3.9%±1.5%, 0.9×10^5^±0.4×10^5^) (**Fig. 5A** **,** **Table 4**). In contrast, we observed significant differences in the frequency of FoxP3^+^CD4^+^T_reg_ cells. Feeding of *B. adolescentis* resulted in a significant increase in FoxP3^+^CD4^+^T_reg_ cell percentages (M (1.7%±0.8%) vs. B (4.8%±1) p<0.05, Y (0.8%±0.3%) vs. BY (4.8%±2.5%) p<0.001, M vs. BY P<0.05, B vs. Y p<0.001) and absolute numbers (M (2.2×10^3^±0.9×10^3^), B (4.9×10^3^±1.3×10^3^) p<0.01, Y (0.7×10^3^±0.4×10^3^) vs. BY (4.2×10^3^±1.3×10^3^) p<0.001, M vs. BY p<0.05, B vs. Y p<0.001) in mice. Furthermore, not even additional infection with *Yersinia* influenced this fact (**Fig. 5B** **,** **Table 4**). In line with the control experiments for pDCs, treatment of mice with streptomycin (S) had no impact on the frequency of FoxP3-positive T cells (M (1.2%±1.1%, 8.7×10^1^±6.5×10^1^) S (0.5%±0.9%, 0.7×10^1^±0.1×10^1^) (**Fig. 3B** **,** **Table 2**). However, depletion of *B. adolescentis* resulted in significantly different percentage and cell count frequency of T_reg_ cells (M +antibiotics (1.5%±0.6%) vs. B + antibiotics (0.5%±0.6%) p = 0.00361; M +antibiotics (3.4×10^3^±2.5×10^3^) vs. B + antibiotics (0.3×10^3^±0.3×10^3^) p = 0.0494) (**Fig. 4B** **,** **Table 3**).

**Table 4:** Counts of Lamina Propria CD4^+^ T cells and FoxP3^+^ Regulatory T Cells.

Colonization	CD3^+^CD4^+^ cells	CD4^+^ FoxP3^+^cells
	cell count ×10^5^	cell count ×10^3^
**M**	1.3±0.7	2.2±0.9^a^
**B**	1±0.4	4.9±1.3
**Y**	0.9±0.5	0.7±0.4^b^
**BY**	0.9±0.4	4.2±1.3

Numbers indicate mean cell counts ± SD of untreated mock (M), *B. adolescentis* fed (B), *Yersinia* infected (Y), as well as *B. adolescentis* fed and *Yersinia* infected mice (BY) mice. ^a^ M vs. B p<0.01, M vs. BY p<0.05, ^b^ B vs. Y p<0.001, BY vs. Y p<0.001. Data represent at least five mice per group.

From the above results, we conclude that feeding of *B. adolescentis* might promote development of T_reg_ cells.

### Plasmacytoid DCs are Crucial for Inhibition of *Y. enterocolitica* Dissemination

In order to examine the inhibitory function of *B. adolescentis*-induced CD11c^int^B220^+^PDCA-1^+^ plasmacytoid (p) DCs on *Y. enterocolitica* dissemination we performed pDC depletion experiments. Therefore we injected *B. adolescentis* fed C57BL/6 mice with anti-mouse PDCA-1 antibody or respective isotype control prior to and during *Yersinia* infection.

**Figure 6:**
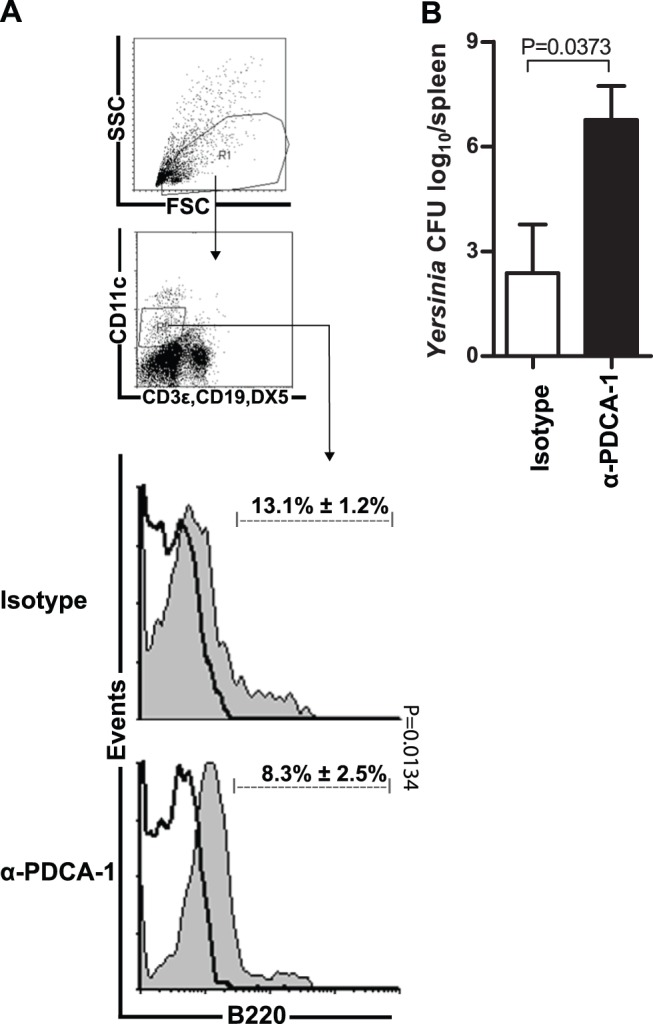
Depletion of pDCs Abrogates *B. adolescentis*-Mediated Prevention of *Yersinia* Dissemination. Lamina propria dendritic cells (lpDC) were analyzed by gating on linage negative (CD3ε, CD19, DX5) and CD11c-intermediate cells, as demonstrated in exemplarily depicted dot plots (**A**) Mean percentage ± SD of B220^+^ lamina propria plasmacytoid DCs (solid black line) and fluorescence minus one control (gray filled histograms) (**B**) and splenic CFU of *Yersinia* in log_10_ per gram of *B. adolescentis* fed and *Yersinia* infected mice, either injected with anti-mouse PDCA-1 (n = 5) or respective isotype control (n = 3).

Successful depletion of pDCs by anti-mouse PDCA-1 antibody injections was verified using flow cytometry analyses. In order to determine pDCs, lpDCs were stained with anti-mouse B220 antibody. Injections of the isotype control had no effect on the proportion of B220^+^ pDCs (13.1%±1.2%, 1.1×10^3^±0.5×10^3^) (**Fig. 6A** **,** **Table 5**) and resulted in same levels as in untreated *B. adolescentis* fed mice (16.8%±1.4%) **Fig. S1**). *B. adolescentis* fed pDC depleted mice, however, showed a significantly reduced frequency of B220^+^ pDCs (8.3%±2.5% p = 0.0134, 0.5×10^3^±0.2×10^3^) (**Fig. 6A** **,** **Table 5**), which was comparable to untreated mock mice (6.8%±1.3%) (**Fig. S1**). Interestingly, we demonstrated that only injections of the anti-mouse PDCA-1 antibody abrogated *B. adolescentis*-mediated prevention of *Yersinia* dissemination whereas the isotype control failed. This fact is indicated by a significantly increased splenic *Yersinia* load of anti-mouse PDCA-1 antibody injected *B. adolescentis* fed mice (CFU log_10_ 6.8±2.2) when compared to isotype control injected *B. adolescentis* fed mice (CFU log_10_ 2.4±2.4, p = 0.0373) (**Fig. 6B**).

**Table 5:** Percentages and Cell Counts of Lamina Propria CD11c^int^ DCs and B220^+^ Plasmacytoid DCs.

Injected antibody	CD11c^int^cells		B220^+^cells
	% leukocytes	cell count ×10^3^	cell count ×10^3^
**Isotype**	1.1±0.1^a^	8.1±3.7	1.1±0.5
**α-PDCA-1**	0.6±0.2	6.2±2.7	0.5±0.2

Numbers indicate mean percentages ± SD and mean cell counts ± SD of *B. adolescentis* fed and *Yersinia* infected mice, either injected with anti-mouse PDCA-1 (n = 5) or respective isotype control (n = 3). ^a^ p = 0.0072.

Based on the presented results we hypothesize that feeding of *B. adolescentis* might lead, by a yet unknown mechanism, to recruitment or increased onsite development of pDCs, which might promote development of regulatory T cells. We suggest that this immune response favors intestinal homoeostasis rather than inflammation, strengthens the intestinal barrier and thereby contributes to inhibition of *Yersinia enterocolitica* dissemination.

## Discussion

Commensals are thought to modulate the susceptibility of the host towards intestinal infections. We recently showed that commensal *B. adolescentis* protects mice from *Yersinia enterocolitica* infection [33]. In line with these results the present study demonstrates that *B. adolescentis*-mediated protection is associated with increased frequency of T_reg_ cells and pDCs. This DC subset seems to be crucial for protection since depletion of pDCs abrogated protection.

The intestinal microbiota exerts a variety of functions thereby strengthen the host resistance against intestinal infections. The high bacterial density and diversity in the mammalian intestine contributes to the prevention from infections with opportunistic and strict pathogens. This mechanism is known as colonization resistance and disturbances of microbial composition leads to an increased susceptibility to e.g. enteric infections [36] or *Clostridium difficile*. [37]. However, in this study colonization resistance seems not to be the underlying mechanism of *B. adolescentis*-mediated protection since *Yersinia* loads in Peyer's patches (PP) and fecal samples were comparable between groups.

In addition, the metabolism of the host depends on the microbiota to degrade otherwise indigestible nutritional carbohydrates which results in the release of short chain fatty acids providing an additional energy source. [38]. Metabolic byproducts fulfill additional tasks indicated by the fact that released short chain fatty acids attenuate intestinal inflammation [39] and protect from enteropathogenic infection [40] in mice.

Moreover, commensals are important to maintain and increase the hosts intestinal epithelial barrier function which helps to defend invasive pathogens. In intestinal epithelial cells (IEC) the recognition of commensal components and metabolites can result in the induction of tight junctions [41], [42], cell growth [43], as well as mucus production [44], [45]. Furthermore, the presence of commensals leads to secretion of antimicrobial compounds by IEC [46] enhancing the intestinal innate immune response to pathogens [13], [47]. In addition to the host, bacteria secrete antimicrobial compounds themselves, termed bacteriocins, able to change the microbial composition [48]. The presence of *B. adolescentis* might protect from Yersiniosis owing to an increase in epithelial barrier function either by direct interactions with IEC or by alterations of the microbiota composition.

The host's resistance to pathogens relies on a fully functional gastrointestinal immune system, which develops from an immature state only after microbiota acquisition [49]. Moreover, the microbiota complexity directly influences intestinal dendritic cell subset composition. A less diverse microbiota resulted in reduced frequency of plasmacytoid dendritic cells in lymphoid tissues [50] and administration of the probiotic preparation VSL#3 decreased the pDC content in the lamina propria of mice. Our results additionally proof the influence of the microbiota diversity on DC composition since *B. adolescentis* feeding was associated with an increase in pDCs. These findings suggest that bacterial compounds might directly induce on-site development or the recruitment of plasmacytoid DCs into the intestinal compartment. The rise in pDCs might depend on invariant NKT (iNKT) cells since a recent study demonstrated that iNKT cells are responsible for the recruitment of pDCs to the pancreas during lymphocytic choriomeningitis virus infection [51]. In this study the release of glycolipids by *B. adolescentis* might result in the activation of iNKT cells which in turn may initiate the recruitment of pDCs to the intestine. The crucial function of pDCs is demonstrated by abolished *B. adolescentis*-mediated protection during Yersiniosis after depletion of pDCs. Depending on location the secretion of type I interferons (IFN) by pDCs can differ, since murine splenic pDCs secret high amounts of IFN-α upon TLR9 ligand stimulation whereas pDC of PP don't. In addition, treatment of splenic pDCs with IL-10, prostaglandine E2, and TGF-β, present at mucosal sites, prevents IFN induction, indicating that the mucosal microenvironment might condition pDCs for poor Type I IFN production [52]. In contrast, peripheral blood pDCs of IBD patients are impaired to secret IFN-α and simultaneously secret increased amounts of pro-inflammatory cytokines after TLR9 ligand challenge [30]. Moreover, IBD patients exhibit an increased frequency of pDCs in the intestinal mucosa when compared to controls [30]. A rise in pDC frequency may be supposed to attenuate inflammation which might be insufficient owing to reduced IFN-α secretion. In line with this is the fact that type I interferons secreted by DCs ameliorate DSS-induced colonic injury and inflammation in mice [53] and that *ifnar^−/−^* deficient mice are more susceptible to DSS [53], [54]. However, IFN-β seems not to be protective in general since administration of IFN-β producing *Lactobacilli* to mice exacerbates DSS-induced disease [54]. In addition, might result the *B. adolescentis*-mediated increase in pDCs in a decreased migration rate of CD103-positive DC to the mesenteric lymph nodes due to a reduced expression of CCR7. Since, in vitro stimulated bone marrow-derived DC exhibited a reduced expression of CCR7 after stimulation with cytokines when IFN-β was present [55]. CD103-expressing DC were shown to be responsible for the transport of living *Salmonella typhimurium* cells to the mesenteric lymph nodes in a CCR7 dependent manner [26]. Moreover, a recent publication demonstrated that CD103-positive DC are able to sample, in addition to CX_3_CR1 expressing DC, luminal *Salmonella typhimurium* cells by the production of transepithelial dendrites [56]. In this study *Y. enterocolitica* might also be transported by CD103-expressing DC to the mesenteric lymph nodes. Therefore *B. adolescentis*-induced pDCs might secret IFN-β which act on CD103-epxressing cells resulting in a lower expression of CCR7 and a decreased migration rate to the mesenteric lymph nodes. This in turn might prevent the transport of *Y. enterocolitica* to the mesenteric lymph nodes.

The potential of pDCs to induce regulatory T (_Treg_) cells was demonstrated for human [35] and murine cells [57]. Constitutively present T_reg_ are essential for the maintenance of intestinal homeostasis and to control inflammation [58]. Especially during inflammation the immunosuppressive function of T_reg_ is crucial since a great deal of intestinal pathology originates not from the infection, instead is owing to an overwhelming immune response [59]. In addition, T_reg_ cells are of importance since inflammation enhances the ability of enteric pathogens to establish infection [60]. Moreover, human IL-10 prevented pathology in *H. pylori* infected *il-10*-deficient mice and is accompanied by an increase in T_reg_ cells [61]. In contrast, during *H. pylori* infection T_reg_ cells can be of disadvantage since *H. pylori* manipulates DCs to become tolerant resulting in the induction of regulatory T cells which dampen the immune response and therefore enable long-lasting gastric colonization [62]. Furthermore, T_reg_ cells are important in parasitic infections given that *Toxoplasma gondii* infection-induced pathology [63] and *Schistosoma mansoni*-caused colonic granulomatous [64] pathology are attenuated by T_reg_ cells. *B. adolescentis* feeding-induced T_reg_ cells might dampen intestinal inflammation resulting in unfavorable environmental conditions for *Y. enterocolitica* to establish a systemic infection. *Yersinia pseudotuberculosis* was demonstrated to disseminate directly from the intestinal lumen to the spleen and not to travel via PP and mesenteric lymph nodes [65]. In this study reduced inflammation caused by T_reg_ cells might maintain intestinal barrier integrity and therefore prevent *Y. enterocolitica* dissemination.

Whether *B. adolescentis* directly interacts with the host mucosal immune system, or whether an indirect effect of *B. adolescentis* reduces the host susceptibility towards *Yersinia* infection by e.g. alteration of the microbiota remains to be elucidated.

Our data provide evidence that commensal *B. adolescentis* increases the frequency of pDC and T_reg_ cells and that this might be essential for host resistance to intestinal infection.

## Supporting Information

Figure S1 *B. adolescentis* Feeding Results in Increased Plasmacytoid DC Frequency. (**A**) Lamina propria dendritic cells (lpDCs) were analyzed by gating on Intraepithelial dendritic cells (ieDC) were analyzed by gating on linage negative (CD3ε, CD19, DX5) and CD11c-positive cells, as demonstrated in exemplarily depicted dot plots. Graph represents mean percentage ± SEM of CD11c^+^ lamina propria leukocytes of untreated mock (M, dark gray bar), *B. adolescentis* fed (B, white bar), *Yersinia* infected (Y, black bar), as well as *B. adolescentis* fed and *Yersinia* infected mice (BY, gray bar). (**B**) Histograms represent mean percentage ± SD of CD11b-expressing conventional lpDCs of untreated mock (M), *B. adolescentis* fed (B), *Yersinia* infected (Y), as well as *B. adolescentis* fed and *Yersinia* infected mice (BY) (solid black line) and respective fluorescence minus one control (gray filled histogram). (**C**) CD11c^int^ lamina propria dendritic cells (lpDCs) were analyzed by gating on Intraepithelial dendritic cells (ieDC) were analyzed by gating on linage negative (CD3ε, CD19, DX5) and CD11c^int^-positive cells, as demonstrated in exemplarily depicted dot plots. Graph represents mean percentage ± SEM of CD11c^int^ lamina propria leukocytes of untreated mock (M, dark gray bar), *B. adolescentis* fed (B, white bar), *Yersinia* infected (Y, black bar), as well as *B. adolescentis* fed and *Yersinia* infected mice (BY, gray bar). Histograms represent mean percentage ± SD of (**D**) B220-expressing and (**E**) PDCA-1-expressing plasmacytoid lpDCs of mock (M), *B. adolescentis* fed (B), *Yersinia* infected (Y), as well as *B. adolescentis* fed and *Yersinia* infected mice (BY) (solid black line) and respective fluorescence minus one control (gray filled histogram). Data represent at least five mice per group.
(EPS)

Table S1 Cell Counts of Total Lamina Propria DCs and cDCs (CD11b^+^CD11c^+^).
(DOC)

Table S2 Cell Counts of Total Lamina Propria CD11c^int^ DCs and pDCs (CD11c^int^ B220^+^ or mPDCA1^+^).
(DOC)
