# Supplementary material for: Plasmacytoid Dendritic Cells Are Crucial in Bifidobacterium adolescentis-Mediated Inhibition of Yersinia enterocolitica Infection
Source: PLoS One. 2013 Aug 20;8(8):e71338. doi: 10.1371/journal.pone.0071338 (PMC3748105; doi:10.1371/journal.pone.0071338)
Supplement: Table S1 — Cell Counts of Total Lamina Propria DCs and cDCs (CD11b+CD11c+). (DOC) [file pone.0071338.s002.doc]

**Supplementary Table S1: Cell Counts of Total Lamina Propria DCs and cDCs (CD11b^+^CD11c^+^)**

| **Colonization** | **CD11c^+^ cells** | | **Conventional** |
| --- | --- | --- | --- |
|  | **cell count x10^4^** | **cell count x10^4^** | |
| **M** | 2.2 ± 0.4 | 1.5 ± 0.2 | |
| **B** | 2.6 ± 0.7 | 1.5 ± 0.7 | |
| **Y** | 5.3 ± 1.9^a^ | 3.7 ± 1^b^ | |
| **BY** | 3.1 ± 0.6 | 1.8 ± 0.6 | |

Numbers indicate mean mean cell counts ± SD of untreated mock (M), *B. adolescentis* fed (B), *Yersinia* infected (Y), as well as *B. adolescentis* fed and *Yersinia* infected mice (BY) mice. **^a^**Mock vs. Y p<0.001, B vs. Y p<0.01, BY vs. Y p<0.05; ^b^ Mock vs. Y p<0.001, B vs. Y p<0.001, BY vs. Y p<0.01. Data represent at least five mice.
