# Supplementary material for: Plasmacytoid Dendritic Cells Are Crucial in Bifidobacterium adolescentis-Mediated Inhibition of Yersinia enterocolitica Infection
Source: PLoS One. 2013 Aug 20;8(8):e71338. doi: 10.1371/journal.pone.0071338 (PMC3748105; doi:10.1371/journal.pone.0071338)
Supplement: Table S2 — Cell Counts of Total Lamina Propria CD11cint DCs and pDCs (CD11cint B220+ or mPDCA1+). (DOC) [file pone.0071338.s003.doc]

**Supplementary Table 2: Cell Counts of Total Lamina Propria CD11c^int^ DCs and pDCs (CD11c^int^ B220^+^ or mPDCA1^+^)**

| **Colonization** | **CD11c^int^cells** | **B220^+^cells** | **mPDCA1^+^cells** |
| --- | --- | --- | --- |
|  | **cell count x10^4^** | **cell count x10^3^** | **cell count x10^3^** |
| **M** | 1.6 ± 0.6 | 1.2± 0.4^b^ | 0.5± 0.3^c^ |
| **B** | 2.2 ± 0.3 | 3.6 ± 0.7 | 2 ± 0.5 |
| **Y** | 4.8 ± 1^a^ | 3.2 ± 0.7 | 1.4 ± 1.1 |
| **BY** | 2.7± 0.5 | 4.6 ± 2.5 | 1.8 ± 0.5 |

Numbers indicate mean percentages ± SD and mean cell counts ± SD of untreated mock, *B. adolescentis* fed (B), *Yersinia* infected (Y), as well as *B. adolescentis* fed and *Yersinia* infected mice (BY) mice. ^a^ Mock vs. Y p<0.001, B vs. Y p<0.001, Y vs. BY p<0.001; ^b^ Mock vs. B p<0.05, Mock vs. BY p<0.01; ^c^ Mock vs. B p<0.01, Mock vs. BY p<0.05. Data represent at least five mice.
